# HIV-1 subtype-specific drug resistance on dolutegravir-based antiretroviral therapy: protocol for a multicentre study (DTG RESIST)

**DOI:** 10.1136/bmjopen-2024-085819

**Published:** 2024-08-21

**Authors:** Matthias Egger, Mamatha Sauermann, Tom Loosli, Stefanie Hossmann, Selma Riedo, Niko Beerenwinkel, Antoine Jaquet, Albert Minga, Jeremy Ross, Jennifer Giandhari, Roger D Kouyos, Richard Lessells

**Affiliations:** 1 Institute of Social & Preventive Medicine, University of Bern, Bern, Switzerland; 2 Centre for Infectious Disease Epidemiology and Research, University of Cape Town Faculty of Health Sciences, Cape Town, Western Cape, South Africa; 3 Department of Infectious Diseases and Hospital Epidemiology, University Hospital Zurich, Zurich, Switzerland; 4 Institute of Medical Virology, University of Zurich, Zurich, Switzerland; 5 Department of Biosystems Science and Engineering, ETH Zürich, Basel, Switzerland; 6 National Institute for Health and Medical Research (INSERM) UMR 1219, Research Institute for Sustainable Development (IRD) EMR 271, Bordeaux Population Health Centre, University of Bordeaux, Bordeaux, France; 7 Centre Médical de Suivi des Donneurs de Sang, Abidjan, Côte d'Ivoire; 8 TREAT Asia/amfAR – The Foundation for AIDS Research, Bangkok, Thailand; 9 KwaZulu-Natal Research Innovation and Sequencing Platform (KRISP), University of KwaZulu-Natal, Durban, South Africa; 10 Centre for the Aids Programme of Research in South Africa (CAPRISA), Durban, South Africa

**Keywords:** adolescent, HIV & AIDS, international health services, drug utilization, public health

## Abstract

**Abstract:**

**Introduction:**

HIV drug resistance poses a challenge to the United Nation’s goal of ending the HIV/AIDS epidemic. The integrase strand transfer inhibitor (InSTI) dolutegravir, which has a higher resistance barrier, was endorsed by the WHO in 2019 for first-line, second-line and third-line antiretroviral therapy (ART). This multiplicity of roles of dolutegravir in ART may facilitate the emergence of dolutegravir resistance.

**Methods and analysis:**

Nested within the International epidemiology Databases to Evaluate AIDS (IeDEA), DTG RESIST is a multicentre study of adults and adolescents living with HIV in sub-Saharan Africa, Asia, and South and Central America who experienced virological failure on dolutegravir-based ART. At the time of virological failure, whole blood will be collected and processed to prepare plasma or dried blood spots. Laboratories in Durban, Mexico City and Bangkok will perform genotyping. Analyses will focus on (1) individuals who experienced virological failure on dolutegravir and (2) those who started or switched to such a regimen and were at risk of virological failure. For population (1), the outcome will be any InSTI drug resistance mutations, and for population (2) virological failure is defined as a viral load >1000 copies/mL. Phenotypic testing will focus on non-B subtype viruses with major InSTI resistance mutations. Bayesian evolutionary models will explore and predict treatment failure genotypes. The study will have intermediate statistical power to detect differences in resistance mutation prevalence between major HIV-1 subtypes; ample power to identify risk factors for virological failure and limited power for analysing factors associated with individual InSTI drug resistance mutations.

**Ethics and dissemination:**

The research protocol was approved by the Biomedical Research Ethics Committee at the University of KwaZulu-Natal, South Africa and the Ethics Committee of the Canton of Bern, Switzerland. All sites participate in International epidemiology Databases to Evaluate AIDS and have obtained ethics approval from their local ethics committee to collect additional data.

**Trial registration number:**

NCT06285110.

**STRENGTHS AND LIMITATIONS OF THIS STUDY**

- DTG RESIST is a large international study of dolutegravir resistance in individuals experiencing virological failure on antiretroviral therapy (ART) in diverse settings in sub-Saharan Africa, Asia and Latin America.
- Sanger and whole genome sequencing will be performed in regional laboratories in Mexico, South Africa and Thailand.
- Genome-Wide Association Study methods, Bayesian evolutionary models and phenotypic testing will explore and predict treatment failure genotypes and their clinical relevance.
- A limitation is the absence of genotypic resistance data from participants before they started dolutegravir-based ART.
- The distribution of HIV-1 subtypes across participating sites is uncertain, and the statistical power of some comparisons of drug resistance mutations will be limited.

## Introduction

 The emergence of HIV drug resistance threatens the United Nations’ goal of ending the HIV/AIDS epidemic by 2030.^1^,^3^ HIV drug resistance has increased substantially with the expansion of access to antiretroviral therapy (ART) in low-income and middle-income countries.^4 5^ The regimen used widely in recent years combined the nucleoside reverse transcriptase inhibitors (NRTIs) tenofovir and emtricitabine with the non-NRTI (NNRTI) efavirenz and thus contained three drugs with a low genetic barrier to resistance. As a result, acquired dual-class NRTI and NNRTI resistance was detected in many people living with HIV (PWH) who experienced virological failure on this regimen.^4^ There has also been a steady increase in pretreatment drug resistance (PDR). By 2016, NNRTI PDR exceeded 10% in Southern and Eastern Africa and approached this level in Latin America and the Caribbean. The 10% NNRTI PDR threshold is the point at which the WHO recommends a change in the standard first-line ART regimen.^6 7^

As a response to the high levels of resistance, WHO endorsed the integrase strand transfer inhibitor (InSTI) dolutegravir in 2019 as the primary choice for both first-line and second-line ART across all groups, including pregnant women and those who may become pregnant.^8^ By mid-2022, over 100 countries had incorporated dolutegravir into their treatment guidelines.^9^ Dolutegravir has a high genetic barrier to resistance,^10 11^ and thus far, only a few people with HIV (PWH) have been found to develop resistance to it.^12^,^14^ The types of mutations that cause dolutegravir resistance may vary by treatment history and HIV-1 subtype. For example, the Arg263Lys mutation appears to be relatively more common in PWH who had not previously received first-generation InSTIs than in those previously exposed to INSTIs.^15^ The Gln148His/Lys/Arg mutation may be less common in subtype C than in other subtypes.^16^

The risk factors and mutation patterns contributing to dolutegravir resistance are not as well defined as those for older antiretroviral drugs. Dolutegravir’s extensive use in areas with limited resources, where treatment options are more uniform, the reuse of drugs is common and access to adherence support, viral load (VL) and resistance testing may be limited, could facilitate the emergence of resistance mutations. The Dolutegravir Resistance (DTG RESIST) study aims to document the resistance patterns and contexts in which dolutegravir resistance emerges and thus contribute to safeguarding the long-term sustainability of the global HIV response. DTG RESIST has two parts. The first is a collaborative analysis of existing data from HIV cohort studies, which has recently been published.^15^ Here, we describe the second part of the study, which prospectively recruits PWH who experience virological failure on dolutegravir-based ART in 17 countries in sub-Saharan Africa, South and Central America, and Asia.

## Study aims and hypotheses

The prospective part of DTG RESIST has three aims:

- In aim 1, we examine viral sequences to determine the prevalence and patterns of dolutegravir resistance in adults and adolescents living with HIV-1.
- In aim 2, we combine the HIV drug resistance data from aim 1 with clinical and programmatic data collected within the International epidemiology Databases to Evaluate AIDS (IeDEA) network to explore the factors associated with emergent dolutegravir resistance.
- In aim 3, we characterise the phenotypic effect of novel drug resistance mutations (DRMs) or novel combinations of mutations and explore the mutational pathways of dolutegravir resistance across HIV-1 subtypes.

We hypothesise that the prevalence and spectrum of InSTI DRMs will differ by HIV-1 subtype and by treatment context (aim 1); that NRTI resistance, previous exposure to InSTI drugs, male sex, younger age, second-line or third-line ART or advanced disease, exposure to rifampicin and lack of routine VL monitoring are risk factors for virological failure and the development of dolutegravir resistance (aim 2); and that HIV genotypes from patients experiencing failure of dolutegravir-based ART are associated with phenotypic resistance. We further hypothesise that these mutations develop along pathways specific to each HIV-1 subtype and, in combination, may compensate for the fitness costs of resistance mutations, that is, restore replication capacity compared with single mutations (aim 3).

## Methods and analysis

**Figure 1:**
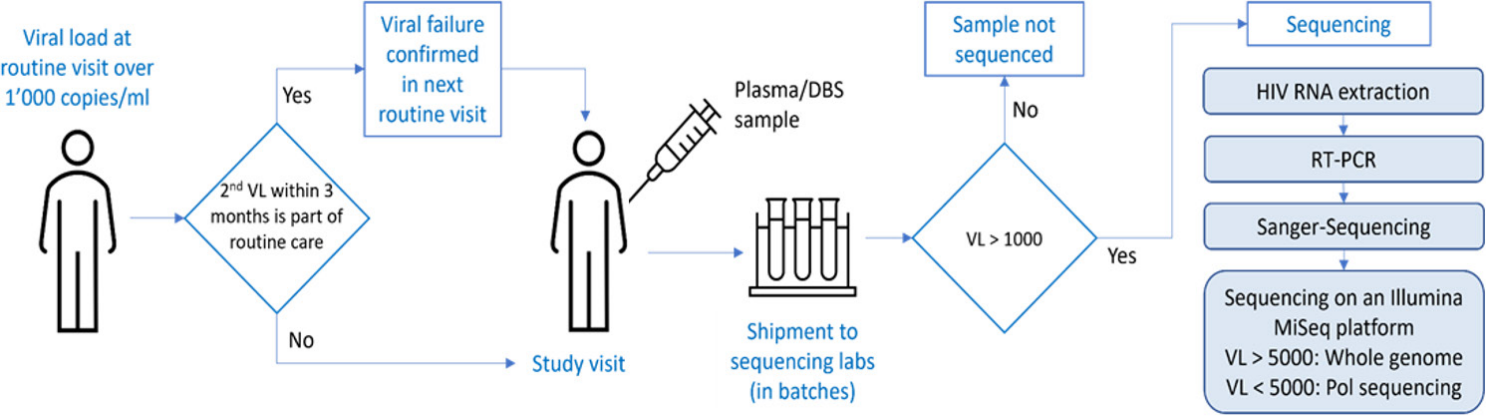
Schematic representation of enrolment, specimen collection and sequencing. DBS, dried blood spot; VL, viral load.

DTG RESIST is a multicentre study enrolling PWH from sub-Saharan Africa, Asia, and South and Central America. Figure 1 summarises the study design. PWH who experience virological failure in routine care (confirmed or based on a single measurement) are enrolled in a dedicated study visit. At the study visit, they provide informed consent and a blood sample, which undergoes sequencing if the VL is above 1000 copies/mL. The DTG RESIST data are linked with the routine data collected within the IeDEA.

### Setting

**Figure 2:**
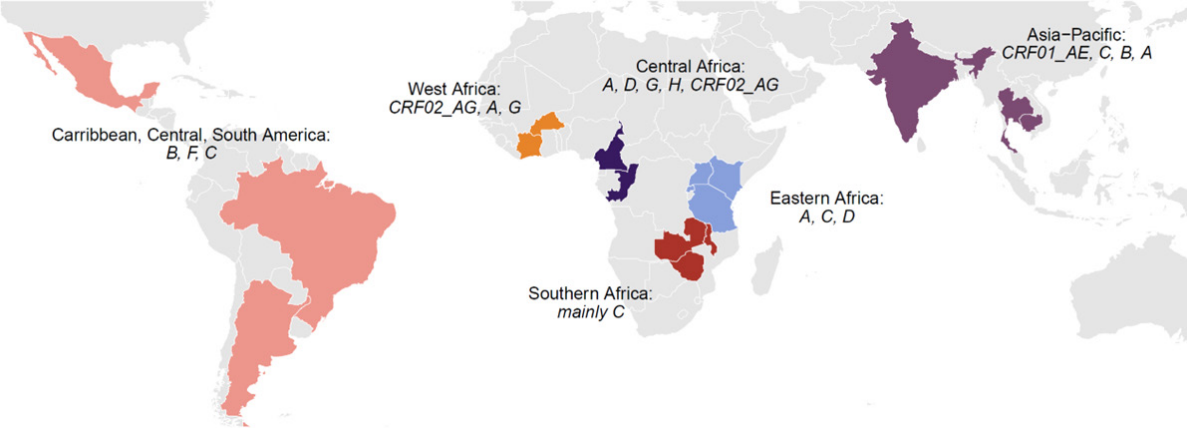
Countries with sites participating in DTG RESIST with the dominant HIV-1 subtypes and circulating recombinant forms. The colours represent the six IeDEA regions. IeDEA, International epidemiology Databases to Evaluate AIDS.

DTG RESIST is embedded within IeDEA. Established in 2006 by the National Institute of Allergy and Infectious Diseases, IeDEA is a global cohort collaboration that collects HIV/AIDS data from HIV care and treatment programmes. Six of the seven IeDEA regions participate in DTG RESIST: Southern Africa, East Africa, Central Africa, West Africa, Asia–Pacific, and the Caribbean, Central and South American region. The IeDEA consortium has been described in detail elsewhere.^17^,^19^ Figure 2 shows the countries participating in DTG RESIST, the HIV-1 subtypes and the Circulating Recombinant Forms (CRFs) prevalent in the region.

### Study population, eligibility and recruitment

DTG RESIST enrols adults and adolescents (age 10–19 years) living with HIV who have been on dolutegravir for at least 3 months and experience virological failure during routine care, defined as a single or, if available, a confirmed VL >1000 copies/mL on any dolutegravir-based combination ART regimen (figure 2). At enrolment, written informed consent is obtained from the participant or the parents or caretaker, as per local regulations. Key clinical and demographic data are collected using the Research Electronic Data Capture system.^20^ The DTG RESIST data will be combined with the clinical and epidemiological data prospectively collected within IeDEA.^17^,^19^

### Specimen collection and transport

Specimen collection and processing are performed according to WHO/HIVResNet guidance.^21^ Whole blood is collected from participants in EDTA tubes. Specimens are kept at room temperature (15°C–30°C) until centrifugation and separation (within 6 hours). All initial processing, centrifugation, pipetting and aliquoting will follow standard laboratory biosafety precautions at a local laboratory equipped to manipulate infectious clinical specimens. Following separation, 4×1.5 mL plasma cryovials from each participant will be frozen at −80°C. Two dried blood spot (DBS) cards with five spots each are also collected and stored at −20°C. In sites where storage of plasma samples is not possible, only DBS cards will be prepared^22^ (figure 1). A courier service experienced in transporting clinical specimens ships plasma and DBS specimens on dry ice to the genotyping labs in Durban, Mexico City or Bangkok.

### Sequencing and phenotypic resistance testing

The KwaZulu-Natal Research Innovation and Sequencing Platform in Durban, South Africa,^23^ the Centro de Investigación en Enfermedades Infecciosas (CIENI), Mexico City^24^ and the HIV-NAT virology lab at Chulalongkorn University, Bangkok, Thailand^25^ will serve as the regional laboratories. Whenever possible, whole HIV-1 genomes using the Illumina MiSeq Next-Generation Sequencing platform will be generated to examine mutations in genetic regions outside pol that could be associated with resistance to InSTIs (figure 1).^26 27^ Alternatively, if low virus loads do not allow whole viral genome sequencing, partial pol sequences, including the viral protease, reverse transcriptase and integrase genes, will be generated. We will perform phenotypic testing on about 100 selected samples focusing on non-B subtype viruses with major InSTI resistance mutations,^28^ including sequences with complex patterns of DRMs. Phenotypic resistance testing will be performed using the PhenoSense Integrase assay (Monogram Biosciences, San Francisco, California, USA^29^), which provides an in vitro measurement of the half maximal inhibitory concentration (IC50) to dolutegravir and the viral replication capacity. To test the reliability of genotypic prediction, we will compare the IC50 with the genotypic sensitivity scores predicted from the Stanford HIVdb, REGA or ANRS algorithms.^30^ We will use a viral Genome-Wide Association Study (GWAS) approach to explore the effect of mutations outside of the integrase.

### Outcomes and statistical analyses

We define two analysis populations to address aim 1 and aim 2: (1) all individuals who experienced virological failure on a dolutegravir-based first-line, second-line or third-line regimen in one of the participating cohorts and (2) all who started ART on a dolutegravir-based regimen or switched to such a regimen and were at risk of developing virological failure (the source population for (1)). The outcome of interest in population (1) will be any InSTI DRMs whereas in (2), it will be virological failure as defined above.

We will analyse population (1) using random-intercept logistic regression and population (2) using parametric survival models,^31^ stratified by treatment programme or country to account for heterogeneity across sites. We will measure the time from the date of switching to DTG-based ART (or from 6 months after initiating DTG-based first-line ART) to the earliest onset of virological failure, date of death or date of last follow-up visit. Exposure variables include age, sex, type of dolutegravir-based ART (initiated first line, switched to first line, second line or third line), previous exposure to InSTI drugs, pre-existing resistance mutations (from HIV-1 genotypes or predicted by the approach described below), advanced disease (as indexed by CD4 count <200 cells/µL), exposure to rifampicin, a history of ART interruptions and VL monitoring coverage and frequency (using approaches used in previous analyses^32 33^). We will follow recommendations on the conduct and reporting of prognostic research, including the choice of variables for inclusion in models and the handling of continuous variables and non-linear relationships.^34^ Missing values will be imputed using multiple imputations, assuming data are missing at random.^35^

We will explore using conjunctive Bayesian network models to assess the mutational pathways of DTG resistance (aim 3).^36^,^38^ The order of mutation accumulation and interdependence between mutations (ie, whether the acquisition of one mutation is less or more likely in the presence of other mutations) will be inferred from the co-occurrence patterns of mutations. We will learn these models for each subtype separately to identify subtype-specific mutational pathways. In exploratory analyses, we will search for novel resistance mutations that may mediate treatment failure and assess the impact of previously proposed mutations, such as mutations outside the HIV-1 integrase.^39 40^ We will combine the genotype data generated in aim 1 with viral whole genome sequences from phylogenetically matched controls from the Los Alamos HIV sequence database. In this pooled dataset, we will use GWAS methodology (as implemented in the PLINK software^41^) to identify the amino-acid mutations associated with failure.

### Sample size and power calculations

Based on a survey of participating sites and recruitment so far, we estimate that about 1500 PWH will experience virological failure, including 900 on first-line and 600 patients on second-line or third-line ART. The number of individuals with DRMs will depend on the distribution across groups of patients initiating or switching to different lines of dolutegravir-based ART, the proportion switching with detectable VL and other factors.

**Figure 3:**
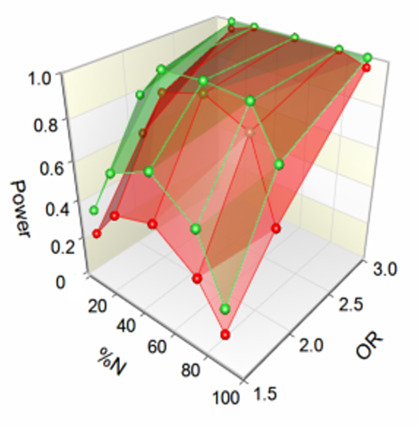
Power versus exposure prevalence (%N) and OR for 84 (red) and 157 (green) patients developing InSTI DRMs. InSTI, integrase strand transfer inhibitor; DRMs, drug-resistance mutations.

For aim 1, we will have reasonable power to detect differences between HIV-1 subtypes. For example, comparing subtype C with subtype B and assuming a total sample of 250 in each group will allow us to detect a difference in the prevalence of resistance mutations of 3% vs 9% with 81% power. The test statistic used for these calculations (done in PASS 15^42^) is the two-sided Z-Test; the significance level of the test is 0.0500. The power will be lower for comparisons with other subtypes with fewer patients. For aim 2, with 1500 patients, we will have ample power to identify risk factors for virological failure in univariable and multivariable models. Power will be more limited for analyses of factors associated with the presence of InSTI DRMs. Assuming that 10% of patients on first-line ART, 20% on second-line ART and 30% on third-line ART will develop virological failure within 2 years, and further assuming that among patients failing first-line ART 1%–3% will develop InSTI DRMs, whereas 10%–20% of patients failing second-line and 20%–30% of patients failing third-line ART will develop InSTI DRMs, we can expect 84–157 patients with InSTI DRMs. As shown in figure 3 (from PASS 15^42^), in univariable analyses, we will have a power of 80% or above to detect risk factors with an odds ratio of 2 or above for a range of exposure prevalence.^43^ Power will be lower in the multivariable models, which should not include more than 5–10 variables.^44^

The first PWH was recruited into the study on 13 June 2022 in Kanyama, Zambia. At the time of writing, over 750 PWH had been enrolled. Recruitment will continue until the end of May 2025.

### Patient and public involvement

There was no involvement of patients or the public in the development of the research questions or the study methods.

## Discussion

In low-income and middle-income countries, WHO recommends a public health approach to providing and monitoring ART,^45^,^48^ including standardised treatment regimens, simplified patient monitoring and clinical decision-making, population-level surveillance and monitoring of HIV drug resistance.^6 49^ WHO guidelines also recommend dolutegravir in first-line, second-line and third-line ART regimens.^8^ This multiplicity of roles of dolutegravir in the public health approach to ART, combined with limited access to VL and HIV drug resistance testing, may facilitate the emergence of dolutegravir resistance. DTG RESIST is a timely study addressing this threat.

DTG RESIST leverages the unique resource of IeDEA, a large international research consortium and rich resource for globally diverse HIV/AIDS data.^17^,^1950^ This brings four key strengths to the study: (1) pooling of data across a network of cohorts to explore patterns and determinants of a rare event, dolutegravir resistance; (2) inclusion of multiple HIV-1 subtypes across diverse geographical areas; (3) harmonised clinical and drug resistance data across participating sites and (4) combined expertise in the clinical, epidemiological, biological and computational fields required to assess the genotype–phenotype mapping. The many important collaborative publications of IeDEA highlight the strength of pooling data across multiple cohorts and regions.^3251^,^57^

DTG RESIST will generate whole HIV-1 genomes and develop robust laboratory procedures to amplify, sequence and assemble the genomes from the diverse set of HIV-1 strains obtained in the multiregional network.^27^ The spectrum of dolutegravir-selected mutations and their effects on phenotypic susceptibility in non-B subtypes is yet to be fully characterised. HIV-1 main group (group M) has diversified into nine subtypes (A–D, D–H and J–K), six subsubtypes (A1–A4 and F1–F2), multiple CRFs and thousands of unique recombinant forms.^58 59^ Most PWH are infected with non-B subtypes, but much of the evidence about ART outcomes and HIVDR is based on subtype B viruses from Europe and North America. HIV-1 subtype could influence treatment outcomes and the emergence of drug resistance.^60^,^62^ Finally, phenotypic testing of non-B subtype viruses with major InSTI resistance mutations and GWAS to explore the effect of mutations outside of the integrase will likely provide new insights into the relevant drug resistance pathways. Bayesian evolutionary models of accumulating mutations will be used to predict the expected genotype at treatment failure.^36^

There are also limitations to the approach. An important weakness is the lack of genotypic resistance data from before dolutegravir treatment for most participants. We considered collecting and biobanking samples from all people starting dolutegravir-based ART and then having paired samples from pretreatment and at the time of virological failure. However, this was not deemed feasible at most IeDEA sites. We will explore whether these missing baseline genotypes can be imputed by adapting models to predict expected resistance genotypes based on treatment and clinical history. Other challenges include the lack of harmonised data on adherence. There is uncertainty about the distribution of HIV-1 subtypes across sites, and the statistical power of the analyses of patterns and risk factors for dolutegravir resistance will be limited. We will consider pooling our data with those from other related studies to address these issues.^15^ The results of the GWAS and Bayesian network analyses may be subject to overinterpretation due to strong model assumptions and limited sample size. To avoid this, we will qualify these as preliminary and hypothesis-generating studies requiring validation.

## Ethics and dissemination

The research protocol was approved by the Biomedical Research Ethics Committee at the University of KwaZulu-Natal and the Ethics Committee of the Canton of Bern. All sites participate in IeDEA and have approval from their Institutional Review Boards (IRB) or Ethics Committees to contribute data to this research network. In addition, each participating site obtained ethics approval from their local ethics committee to conduct the specific additional data collection, including the determination of adequate provisions solicitation assent by adolescents or parental consent. The research findings will be shared through open-access publications and in dissemination meetings with local stakeholders, healthcare providers and communities.
