# Supplementary material for: HIV-1 subtype-specific drug resistance on dolutegravir-based antiretroviral therapy: protocol for a multicentre study (DTG RESIST)
Source: BMJ Open. 2024 Aug 21;14(8):e085819. doi: 10.1136/bmjopen-2024-085819 (PMC11340720; doi:10.1136/bmjopen-2024-085819)
Supplement: Supplementary file 1 [file bmjopen-2024-085819.reviewer_comments.pdf]

PEER REVIEW HISTORY

BMJ Open publishes all reviews undertaken for accepted manuscripts. Reviewers are asked to complete a checklist review form and are provided with free text boxes to elaborate on their assessment. These free text comments are reproduced below.

ARTICLE DETAILS

**TITLE (PROVISIONAL)**	HIV-1 subtype-specific drug resistance on dolutegravir-based antiretroviral therapy: protocol for a multicentre longitudinal study (DTG RESIST)
**AUTHORS**	Egger, Matthias; Sauermann, Mamatha; Loosli, Tom; Hossmann, Stefanie; Riedo, Selma; Beerenwinkel, Niko; Jaquet, Antoine; Minga, Albert; Ross, J; Giandhari, Jennifer; Kouyos, Roger D.; Lessells, Richard

VERSION 1 - REVIEW

**REVIEWER NAME**	Choudhuri, Indrani
**REVIEWER AFFILIATION**	Temple University
**REVIEWER CONFLICT OF INTEREST**	None
**DATE REVIEW RETURNED**	31-Mar-2024

**GENERAL COMMENTS**	Recommendation: Minor revision is recommended.<br>Comments: The manuscript “HIV-1 subtype-specific drug resistance on dolutegravir based antiretroviral therapy: protocol for a multicentre longitudinal study (DTG RESIST)” by Egger and co-workers performs a multicentre longitudinal study of adults and adolescents living with HIV in sub-Saharan Africa, Asia, and South and Central America who experienced virologic failure on dolutegravir-based ART. They aim to characterize the phenotypic effect of novel drug resistance mutations or novel combinations of mutations, and explore the mutational pathways of dolutegravir resistance across HIV-1 subtypes. There protocol and methodology are mostly thorough and articulate. Thus, I suggest a minor revision and no further revision is needed. Please see the specific comments below.<br>Specific comments:<br>1. The author mentioned that DTG RESIST enrols adults and adolescents (ages 10-17). But WHO defines an adolescent as any person between ages 10 and 19 and adults ≥ 19. It will be helpful if the author can provide some explanation that why they mentioned “adults” in their study.<br>2. It will be helpful if the author can also mention the gender of the patients to get more detailed understanding of the Specimen.

**REVIEWER NAME**	Biswas, Avik
**REVIEWER AFFILIATION**	University of California San Diego
**REVIEWER CONFLICT OF INTEREST**	None
**DATE REVIEW RETURNED**	19-Apr-2024

**GENERAL COMMENTS**	I found the protocol to be highly relevant, comprehensive, well-written, and would be of significant interest to the readership of BMJ. It is my belief that the research study would be of substantial clinical impact, and I support publication of the protocol. My one minor comment is that the authors should clearly indicate, highlight and substantiate what the major new findings of the study would be that would take impact the field of HIV Biology as well as the clinical outcome of patients, and is not available from the prior (extensive) literature on the subject.

VERSION 1 – AUTHOR RESPONSE

Reviewer 1

Dr. Indrani Choudhuri, Temple University

Comments to the Author:

Manuscript id: bmjopen-2024-085819

Recommendation: Minor revision is recommended.

Comments: The manuscript "HIV-1 subtype-specific drug resistance on dolutegravir based antiretroviral therapy: protocol for a multicentre longitudinal study (DTG RESIST)" by Egger and co-workers performs a multicentre longitudinal study of adults and adolescents living with HIV in sub-Saharan Africa, Asia, and South and Central America who experienced virologic failure on dolutegravir-based ART. They aim to characterize the phenotypic effect of novel drug resistance mutations or novel combinations of mutations, and explore the mutational pathways of dolutegravir resistance across HIV-1 subtypes. There protocol and methodology are mostly thorough and articulate. Thus, I suggest a minor revision and no further revision is needed. Please see the specific comments below.

Thank you.

Specific comments:

1. The author mentioned that DTG RESIST enrols adults and adolescents (ages 10-17). But WHO defines an adolescent as any person between ages 10 and 19 and adults ≥ 19. It will be helpful if the author can provide some explanation that why they mentioned "adults" in their study.

Thank you for pointing out this typo. "Adolescent" is now defined as ages 10-19 years.

2. It will be helpful if the author can also mention the gender of the patients to get more detailed understanding of the Specimen.

The final gender distribution of participants is unknown at present. It is clear from the description of the eligibility criteria that participants will be recruited independently of their gender.

Reviewer 2

Dr. Avik Biswas, University of California San Diego

Comments to the Author:

I found the protocol to be highly relevant, comprehensive, well-written, and would be of significant interest to the readership of BMJ. It is my belief that the research study would be of substantial clinical impact, and I support publication of the protocol.

Thank you.

My one minor comment is that the authors should clearly indicate, highlight and substantiate what the major new findings of the study would be that would take impact the field of HIV Biology as well as the clinical outcome of patients, and is not available from the prior (extensive) literature on the subject.

We respectfully disagree with the reviewer. At present, the literature on dolutegravir resistance is limited. In their 2024 HIV drug resistance report, the World Health Organization (WHO) states that "the risk factors and mutation patterns contributing to dolutegravir resistance are not as well-defined as those for older antiretroviral drugs. "

As we argue in the paper, DTG RESIST will document the resistance patterns and contexts in which dolutegravir resistance emerges, characterize the phenotypic effect of novel drug resistance mutations or novel combinations of mutations, and explore the mutational pathways of dolutegravir resistance across HIV-1 subtypes. It will thus make an important contribution to a better understanding of the epidemiology and biology of dolutegravir resistance across a wide range of settings, and contribute to safeguarding the long-term sustainability of ART.

We discuss this point in the last paragraph of the introduction section and the third paragraph of the discussion, as follows:

"The risk factors and mutation patterns contributing to dolutegravir resistance are not as well-defined as those for older antiretroviral drugs. Dolutegravir's extensive use in areas with limited resources, where treatment options are more uniform, the reuse of drugs is common, and access to adherence support, viral load, and resistance testing may be limited, could facilitate the emergence of resistance mutations. The DTG RESIST study aims to document the resistance patterns and contexts in which dolutegravir resistance emerges and thus to contribute to safeguarding the long-term sustainability of the global HIV response."

"DTG RESIST will generate whole HIV-1 genomes and develop robust laboratory procedures to amplify, sequence and assemble the genomes from the diverse set of HIV-1 strains to be included from the multiregional network.[27] The spectrum of dolutegravir-selected mutations and their effects on phenotypic susceptibility in non-B subtypes is yet to be fully characterized. HIV-1 main group (group M) has diversified into nine subtypes (A-D, D-H, J-K), six sub-subtypes (A1-A4, F1-F2), multiple circulating recombinant forms (CRFs) and thousands of unique recombinant forms.[58,59] Most people living with HIV are infected with non-B subtypes but much of the evidence about ART outcomes and HIVDR is based on subtype B viruses from Europe and North America. HIV-1 subtype could influence treatment outcomes and the emergence of drug resistance.[60–62] Finally, phenotypic testing of non-B subtype viruses with major InSTI resistance mutations, and GWAS to explore the effect of mutations outside of the integrase will likely provide new insights into the relevant drug resistance pathways. Bayesian evolutionary models of accumulating mutations will be used to predict the expected genotype at treatment failure.[36] "
